# Blood Banking in Living Droplets

**DOI:** 10.1371/journal.pone.0017530

**Published:** 2011-03-11

**Authors:** Josh Samot, Sangjun Moon, Lei Shao, Xiaohui Zhang, Feng Xu, YoungSeok Song, Hasan Onur Keles, Laura Matloff, Jordan Markel, Utkan Demirci

**Affiliations:** 1 Demirci Bio-Acoustic-MEMS in Medicine (BAMM) Laboratory, Center for Bioengineering, Harvard Medical School, Brigham and Women's Hospital, Boston, Massachusetts, United States of America; 2 Harvard-MIT Division of Health Sciences and Technology, Massachusetts Institute of Technology, Cambridge, Massachusetts, United States of America; Université de Technologie de Compiègne, France

## Abstract

Blood banking has a broad public health impact influencing millions of lives daily. It could potentially benefit from emerging biopreservation technologies. However, although vitrification has shown advantages over traditional cryopreservation techniques, it has not been incorporated into transfusion medicine mainly due to throughput challenges. Here, we present a scalable method that can vitrify red blood cells in microdroplets. This approach enables the vitrification of large volumes of blood in a short amount of time, and makes it a viable and scalable biotechnology tool for blood cryopreservation.

## Introduction

Blood shortages pose a major global health challenge that frequently occur during natural disasters, military conflicts, and in clinical settings due to fluctuations in supply and demand [1]. Long-term cryopreservation of blood products provides a supplementary inventory to help meet the demand during such shortages by freezing excess blood. Although the use of additive preservatives has extended the liquid storage of blood products to several weeks (i.e., 42 days for red blood cells (RBCs) [2], [3], [4]), the limited shelf life makes it difficult to manage blood inventories resulting in a large waste [5]. For instance, in 2006, 1.2 million units of blood were discarded in the US alone [6], [7]. New technologies can potentially revolutionize how blood is handled in war and global disaster zones, prevent waste, and reduce vulnerability to shortages.

Over the last century, significant progress has been made in understanding the basic factors leading to cryoinjury in RBCs and in development of effective techniques to prevent it [5], [8]. Two major clinical RBC cryopreservation approaches have been established: the high glycerol/slow freezing [9], [10] and the low glycerol/rapid freezing [11], [12], [13] techniques. The high glycerol/slow freezing technique uses 40% (w/v) glycerol with a cooling rate of ∼1°C/min and storage at −80°C. The low glycerol/rapid freezing approach uses 15–20% glycerol with rapid cooling rates (60–120°C/min) by immersing samples in freezing containers into liquid nitrogen (−196°C) or nitrogen vapor (−165°C) [1]. However, although both RBC cryopreservation methods are considered effective, cryoinjury to RBCs still occurs during the cooling and warming processes as a result of cell shrinkage [14], [15], toxicity due to the increasing concentrations of solutes [16], [17], [18] during slow freezing, and intracellular ice formation (IIF) during rapid freezing [19]. In contrast, vitrification as a cryopreservation method has provided a means to significantly reduce the damage to various cells and tissues [20], [21], since ice crystal formation and the corresponding intra and extracellular solute accumulation are prevented. Despite the potential advantages of vitrification, its broad application to RBC biopreservation hasn't yet been achieved. Challenges include difficulty to achieve extreme cooling and thawing rates, toxicity of high cryoprotectant agent (CPA) concentrations needed to vitrify suspensions at a realizable temperature, devitrification upon rewarming, and inability to accommodate freezing of large volumes of blood using microliter cryo vials. Here, we introduce a high throughput ultra-rapid vitrification method using cell encapsulating droplets, which could potentially overcome some of the limitations by lowering the required CPA concentrations and achieving ultra-rapid cooling rates via vitrifying RBCs encapsulated in small droplet volumes. Furthermore, RBCs can be stored in liquid nitrogen directly on the collection film. This is the first time that a scalable vitrification method has been introduced to blood cryopreservation. Such technologies have other broad applications in multiple fields, including film boiling [22], spray cooling during the heat treatment of metallic alloys, turbine engines, and nuclear reactors [23].

## Results and Discussion

**Figure 1:**
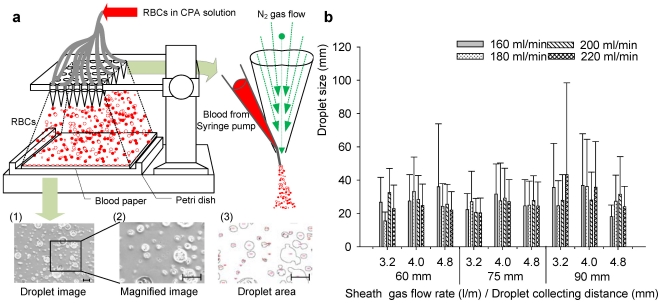
A schematic drawing of the droplet generating system for the blood cryo-preservation process. (**a**) Schematic description for blood droplet ejection on collection film and images of droplets. Scale bar is 500 µm. (**b**) Size distribution of ejected droplets. Average droplet size and standard deviation are shown as a function of droplet collecting distance, sheath gas flow rate, and blood flow rate. Error bars are standard deviations.

It has been shown that the degree of crystallization during ultra-rapid cooling increases with an increase in droplet radius, especially when the dimensionless radii (r*) are above 0.1 (100 µm) [24]. Therefore, we designed a co-flow ejection system to generate RBC encapsulating microdroplets (<100 µm) which can then be vitrified at high throughput. We investigated three major parameters affecting the droplet size, including the droplet collection distance (from ejector tip to droplet collection film), the flow rate of nitrogen gas and the flow rate of CPA loaded RBCs. Droplets generated at different nitrogen flow rates (3.2–4.8 l/min), ejection collecting distances (60–90 mm) were deposited on collection films (**Fig. 1a**) and the droplet size distributions were analyzed (**Fig. 1b**). A decrease in droplet size was observed when either the nitrogen gas flow rate or ejection collecting distance was increased (**Fig. 1b**). However, an increase in flow rate of CPA loaded RBC solution did not result in a significant change in droplet size due to the dominant effect of nitrogen gas flow rate (**Fig. 1b** **, SI Fig. S1b, c, and S2**) within the range from 3.2 to 4.8 l/min over the droplet size. By controlling the nitrogen gas flow rate, droplet diameters were maintained below 100 µm to ensure effective vitrification at a low CPA concentration (2.5 M glycerol, equivalent to a concentration of 23%, **SI Table S1**) where we operated our system minimizing possible toxic and osmotic effects [9], [10].

**Figure 2:**
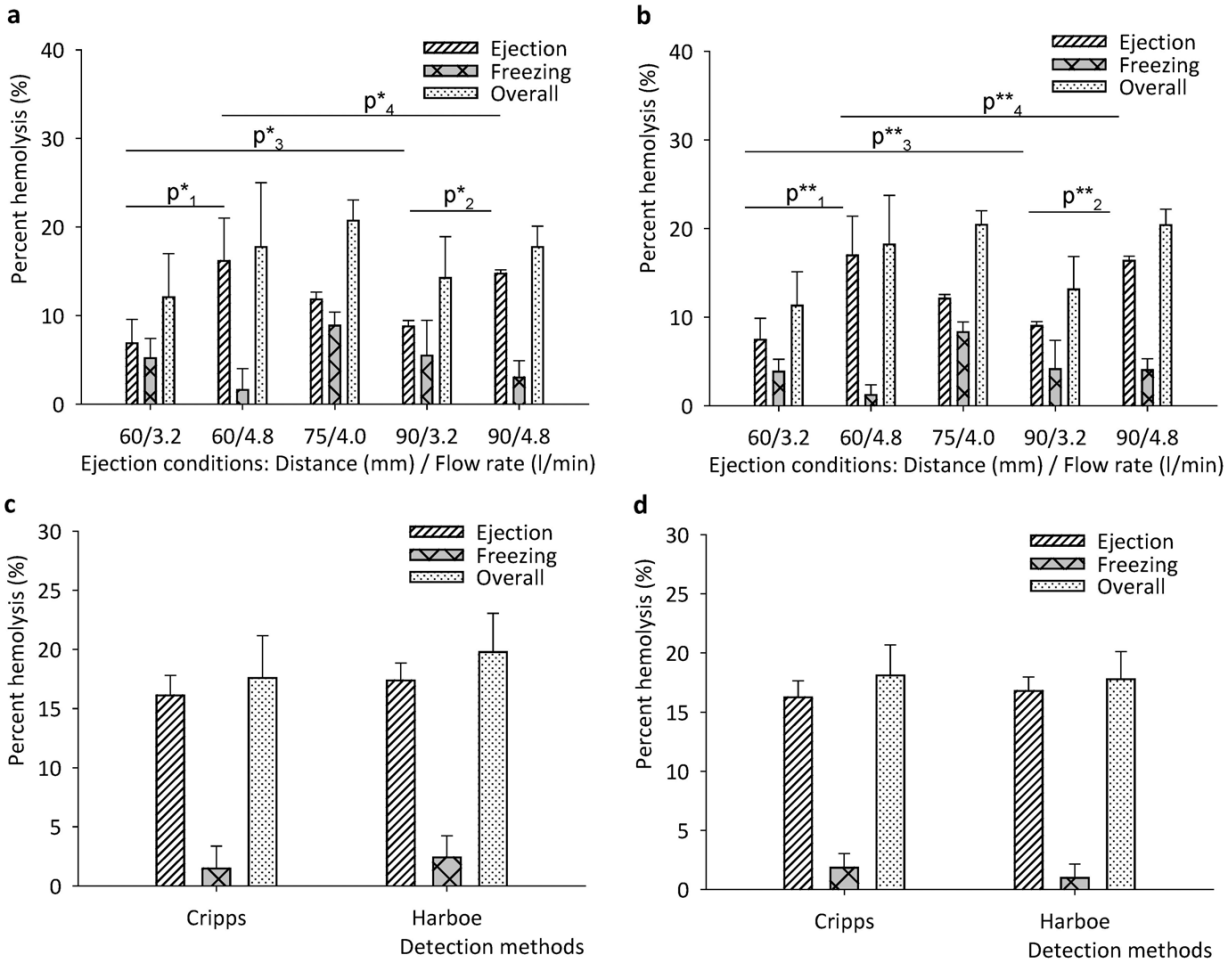
Percent hemolysis values for ejection and freezing at five different conditions (a) Cripps method and (b) Harboe method. P-values were tested at two different distances (60 and 90 mm) and gas flow rates (3.2 and 4.8 l/min), **SI Table S6**. Percent hemolysis values of parallel ejection systems were shown for (**c**) 4 parallel ejectors and (**d**) 25 parallel ejectors system. Both 4 and 25 ejector systems were operated at 3.2 l/min and 6 mm.

To assess overall hemolysis, we evaluated the percent hemolysis from each procedure step including CPA loading, ejection, RBC droplet collection on films, and freezing/thawing at five different experimental conditions (**Fig. 2a**). The percent hemolysis of each step was calculated using **SI Eqn. S1**. with the absorbance values determined using Cripps and Harboe methods as shown in **SI Table S2 and Fig. S3**. We observed that percent hemolysis due to freezing/thawing and shear stress during ejection ranged from 2 to 8% and 5 to 17% across all experimental conditions, respectively (**Fig. 2a**). The CPA loading steps led to a smaller percent hemolysis (2% out of 20% total, **SI Table S2**) compared to the ejection and vitrification/thawing steps. We also observed that, the percent hemolysis by the collection film was negative due to the possible adhesion of free hemoglobin to the film.

The results showed that an increase in droplet collecting distance from 60 mm to 90 mm did not change percent hemolysis either during ejection or freezing/thawing processes at constant gas flow rates of 3.2 l/min and 4.8 l/min, (**Fig. 2a and 2b**). However, keeping the ejection distance constant, when nitrogen gas flow rate was increased from 3.2 l/min to 4.8 l/min, a statistically significant increase in percent hemolysis was observed at ejection distances of 60 mm (increase from 6.9% to 16.16%) and 90 mm (increase from 8.8% to 14.7%) (**SI Table S3 and for statistical analysis: SI Table S6**). These observations indicated that the ejector gas flow rate affects RBC hemolysis (Kruskal-Wallis non-parametric analysis of variance, p<0.05) and damage to cells during droplet generation and encapsulation can be minimized by changing the gas flow rate. For the vitrification and thawing steps, an increase in nitrogen flow rate did not have a significant effect on RBC hemolysis (**Fig. 2a and 2b**) since the average droplet diameter was below 100 µm at each flow rate (**Fig. 1b**). The combined effects of ejection, freezing, and thawing at the lowest nitrogen flow rate (3.2 l/min) resulted in the lowest percent hemolysis (11%) with minimal dependence on ejection distance.

To demonstrate the scalability of the system, we performed droplet-based RBC vitrification experiments using arrays of 4 and 25 independent ejectors activated simultaneously (**SI Fig. S1c**). The vitrification was performed at an ejection distance of 60 mm, CPA loaded RBC flow rate of 0.2 ml/min for each ejector and nitrogen gas flow rate of 3.2 l/min. With this 25-ejector setup, in 5 minutes, the system vitrified 25 ml of RBCs loaded with 2.5 M glycerol (1∶1, v/v). As shown in **Fig. 2c and d**, the percent hemolysis using the 4 and 25 ejector systems were 17.58±3.56% and 18.08±2.59%, respectively. Compared to the values obtained using a single ejector (12.07±4.92% under the same operation conditions, as shown in **SI Table S3**), the use of a multi-ejector system resulted in only a moderate increase to percent hemolysis. The Kruskal-Wallis (non-parametric) analysis of variance on the experimental results indicated that the number of ejectors did not have a significant effect (p>0.05) on the percent hemolysis. Furthermore, pair-wise comparisons with nonparametric Mann-Whitney U test on hemolysis values for single to 4, 4 to 25 and single to 25 ejectors also pointed that the effect of number of ejectors is insignificant with p scores of 0.08, 1.00, and 0.08, respectively (**SI Table S4 and S5**). These results indicated the scalability of the system to process blood at high throughput. Further, for the system to become a clinically useable method, the sterility and avoidance from microbiological contamination are very important in blood transfusion. As a first step taken towards this direction, the ejector system is operated in a sterile hood during ejection, droplet collection and thawing steps.

In summary, we have introduced a scalable vitrification method for the cryopreservation of RBCs by generating microdroplets that are vitrified and thawed at low CPA levels. We envision that the RBC cryopreservation approach presented here has potential to improve the efficiency with which global blood inventories are managed leading to significant economic and social downstream impact.

## Materials and Methods

The RBC cryopreservation process consists of four main steps: blood preparation, CPA loading (**SI Fig. S1a**), ejection, and freezing/thawing/collection (**SI Fig. S1b**). The droplets were generated from the co-flow stream of the CPA-loaded RBC solution and nitrogen gas flow through an ejector (**SI Fig. S1c**). All experiments were performed in a sterile hood to prevent microbacterial contamination that could have adverse affects on the RBCs. All the abbreviations are listed in **SI Table S7**.

### Blood Preparation

All buffy coat samples were received from the Massachusetts General Hospital transfusion center. The buffy coat was easier to obtain compared to whole blood, and prepared specifically for research use. To isolate RBCs, 25 ml of buffy coat sample was first mixed with 3.5 ml of Citrate Phosphate Dextrose Adenine (CPDA-1) anticoagulant for 2 min at 20°C, and then centrifuged at 2000 rpm for 10 min at 20°C (Allegra 6 Centrifuge, Beckman Coulter, USA). After removing the supernatant, approximately 5 ml of RBC pellets remained at the bottom. For each 100 ml of CPDA-1, 327 mg citric acid, 2.635 g sodium citrate, 222 mg monobasic sodium phosphate, 3.175 g dextrose, 27 mg adenine were used.

### CPA Loading

The CPA solutions at different concentrations (1M, 2M, 2.5M and 4M) were prepared, and the compositions were listed in **SI Table S1**. 5 ml of collected RBC pellet was first mixed with 2M glycerol at a ratio of 1∶1 (v/v) (Sigma, USA) to achieve a final glycerol concentration of 1M. The mixture was then centrifuged at 2000 rpm for 10 minutes and the isolated RBCs containing 1M glycerol were collected, and then further mixed with 4M glycerol at a ratio of 1∶1 (v/v) to obtain a final glycerol concentration of 2.5M.

### Cell Encapsulating Droplet Generation System

The droplet generation system is shown in **Fig. 1a** and **SI Fig. S1c**. An ejector was built using a 200 µl pipette tip attached to a 27 gage stainless needle tip (BD Biosciences, San Jose, CA). The needle tip was placed into the center of the pipette tip to build a co-flow nozzle. The two components were assembled by inserting the needle tip across pipette tip wall at a point 2 cm away from the pipette tip end. The needle tip was pushed further inside the pipette tip until it stuck out 2 mm from the edge. A nitrogen gas tank was connected to the pipette tip with Tygon® tubing (ID = 3.2 mm) (Saint-Gobain Performance Plastics, Worcester, MA) through which the gas could flow to the pipette tip (**Supp.** **Fig. 1c**). A CPA loaded RBC sample was loaded into a syringe attached to a 30 gage needle (Small Parts Inc., Miramar, FL). A 15 cm polyethylene tubing (Becton Dickinson Primary Care Diagnostics, Sparks, MD) through which the sample could flow was then used to connect the blood loaded syringe and ejector through the needle tip. The RBC sample loaded with CPAs was then loaded into a syringe pump (World Precision Instrument, Sarasota, FL) Nitrogen gas was flowed through the Tygon tubing to the pipette tip simultaneously while the CPA loaded RBC solution was pumped from the syringe pump and flowed through the polyethylene tubing to the needle tip, resulting in a force that created droplets from the needle tip of the ejector (**Fig. 1a**). The ejector was cleaned with ethanol and autoclaved before use.

### Vitrification of RBCs

The RBC sample loaded with CPAs was delivered to the ejector from the syringe pump at a flow rate of 0.2 ml/min. The nitrogen gas was supplied from the nitrogen gas tank and delivered to the ejector through Tygon® tubing at a rate of 4 l/min. For the vitrification of RBCs, the droplets were first ejected onto a polyethylene (PE) collection film (Avery, Brea, CA) of 8.5 mm diameter. Then, the film with the RBC droplets attached was rapidly immersed into liquid nitrogen using pre-cooled tweezers.

### Thawing and Collection of RBCs

For the thawing, the film with the CPA loaded RBC droplets attached was removed from the liquid nitrogen using tweezers and immediately transferred in a petri dish filled with a thawing solution consisting of 10 ml of 2.5M glycerol pre-warmed to 25°C (**Supp. Fig. S1c**). The film was surrounded by liquid nitrogen vapor during the transfer to prevent any devitrification prior to immersion in the thawing solution. The film with the CPA loaded RBC droplets was immersed completely in the thawing solution and the droplets were washed completely off the film and into the thawing solution. Once the vitrified droplets were completely thawed and the RBCs were washed into the thawing solution, the mixture containing RBCs and 2.5M glycerol was then collected and centrifuged at 2000 RPM for 10 min. The supernatant was then removed and RBCs in 2.5M glycerol solution were collected.

### Droplet size measurement

Since the degree of crystallization of the droplet increases with droplet size, we measured the droplet size distribution at 5 different experimental conditions. Three major variables were investigated for their effects on droplet size distribution, including (i) droplet collection distance between ejector tip and droplet collection film, (ii) flow rate of nitrogen gas, and (iii) the flow rate of CPA loaded RBCs. At each operation condition, droplets were ejected onto the surface of a 150 mm polystyrene petri-dish, and then images were taken using a microscope (TE 2000; Nikon, Japan). The images were taken at 10× magnification, and the number of droplets and their diameters were measured using Image J (NIH, Bethesda). To create an accurate read-out of the droplet diameter, the images were first adjusted to desired threshold level (0∼255 for 8bit gray scale image). Droplets that did not initially appear as closed circles on the image were modified using black/white threshold imaging process and the dot tools of image J. Then the data acquired was transferred to Excel where measurements were converted from pixels to micrometers to obtain droplet size (one pixel = 1.28 µm). Finally, the distribution of droplet size was plotted using Sigmaplot (Systat Software Inc., Chicago, IL). The results were shown in **SI Fig. S2a** and **S2b**.

### Percent Hemolysis Analysis

To better understand how each procedure impacts the final RBC hemolysis after vitrification, we analyzed for the percent hemolysis for each step (i.e., CPA loading, droplet ejection, collecting droplets on film, and freezing/thawing). We calculated the percent hemolysis by comparing the free hemoglobin in solution after each process is performed to those of controls. The controls were the free hemoglobin present in a sample which is prepared before a given step is performed and in a sample which is prepared with DI water for 100% hemolysis value. The first control sample shows how much hemoglobin is already present before a step is performed and the second control shows how much hemoglobin is present when 100% hemolysis occurs. Percent hemolysis can be expressed by the following equation [1]–[5]:
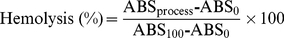
(1)Where 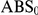 and 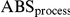 are the absorbance of free hemoglobin in RBC samples before and after each step of the RBC encapsulating droplet cryopreservation process is performed, respectively. 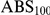 is the absorbance of free hemoglobin in RBC samples after total cell lysis in DI water. The numerator represents the amount of hemoglobin released during a given experimental step while the denominator represents the total amount of hemoglobin present in the RBCs before this step was performed. The fraction of hemoglobin that was intact in the cells is given by, 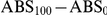, before a given step is performed. The hemoglobin released from the cells while the step was being performed is given by, 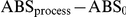. Dividing the numerator by the denominator gives the percentage of hemolysis. Absorbance was measured using an UV-VIS spectrophotometer (UV-2450, SHIMADZU, Japan) as shown in **SI Fig. S3**. Hemolysis was determined using the Cripps and Harboe methods [25], which are two standard methods to calculate hemolysis based on hemoglobin absorbance at different wavelengths. Results of each process step were presented with these spectrophotometer values, **SI Table S2**.

### Hemolysis due to loading of CPA_1_ (addition of 2M CPA to RBC pellet for a final 1M CPA concentration)

To prevent ice crystals from forming in the RBCs during freezing/thawing, CPAs must be loaded to the cells. Before the CPA_1_ loading step was performed, two control samples were prepared to obtain absorbance measurements (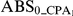 and 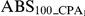). A 20 µl sample of isolated RBCs was added to 10 ml of Dulbecco's Phosphate Buffered Saline (DPBS) (GIBCO, Grand Island, NY) to dilute the RBCs to a concentration within the range of the spectrophotometer reading where free hemoglobin is linearly correlated to the absorbance, for 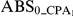 measurement. Since minimal hemolysis occurs during the addition of DPBS, the absorbance value obtained accurately reflects the free hemoglobin concentration before 2M glycerol was loaded. To obtain a RBC sample for 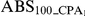 measurement, a 20 µl sample of isolated RBCs was added to 10 ml of deionized (DI) water to obtain a total cell lysis. To perform the first CPA loading step, 1 ml of isolated RBCs was mixed with 2M glycerol with a ratio of 1∶1 (v/v) (Sigma, USA) to obtain a RBC sample with a final glycerol concentration of 1M. Then 40 µl of this CPA loaded sample was mixed with 9.98 ml of 1M glycerol to match the same mixing ratios as the control samples. These three samples were then centrifuged at 2000 rpm for 10 min and the absorbance of the supernatants was measured using the UV-VIS spectrophotometer (UV-2450, Shimadzu, Japan).

### Hemolysis due to loading of CPA_2_ (addition of 4M CPA to RBCs in 1M CPA solution for a final 2.5M CPA concentration)

Two 20 µL samples of isolated RBCs loaded with 1M glycerol were taken into two tubes. One sample was diluted with 10 ml of 1M glycerol, and the other was diluted with 10 ml of DI water to obtain control samples for ). For pairwise comparisons, one-tailed p-value was used to evaluate the effect of ejection distance and gas flow on percent hemolysis; whereas two-tailed p-value was used to evaluate the effect of number of ejectors on percent hemolysis.

## Supporting Information

Figure S1 **Overall schematic of the blood cryopreservation setup and process steps.** (**a**) CPA loading process for whole blood, (**b**) Vitrification process (**c**) Picture of multi-ejector setup, ejector length (L_ext_) and needle tip (D_needle_) was 3.0 mm outer diameter and 210 µm inner diameter (27 gauge needle). Scale bar is 1 mm.
(TIF)

Figure S2 **Droplet size measurements.** (**a**) Images of ejected droplets on collection film. Outline tracking, measuring size, and counting of droplets using Image J software. Droplets were collected on paper with three different distances and sheath flow rates at a fixed blood flow rate, 200 µl/min. Droplet diameters were calculated using the area of each closed outline. (**b**) Droplet size distribution is shown as a function of droplet collecting distance, gas flow rate, and blood flow rate.
(TIF)

Figure S3 **Absorbance values and percent hemolysis for different steps during cryopreservation process.** Percent hemolysis was measured for each process step following **Eqn. S1** as described in the *Percent Hemolysis Analysis* section in the manuscript. Each dotted box represents how each process affects hemolysis, i.e. (**a**) CPA_1_ loading effect (ABS_CPA1_), (**b**) CPA_2_ loading effect (ABS_CPA2_), (**c**) ejection effect (ABS_ejection_), and (**d**) freezing and thawing (ABS_freeze_) effect.
(DOCX)

Table S1 **Composition of the cryoprotective solutions used (g/40 ml).**
(DOC)

Table S2 **Spectrometer absorbance values for the two controls and actual sample from each step in the cryopreservation process from experimental conditions of 75 mm droplet collecting distance and 4.0 l/min of sheath gas flow rate).**
(DOC)

Table S3 **Percent hemolysis values of ejection, collection film, and freezing for five different experimental conditions are given.** Total hemolysis is the sum of hemolysis due to the ejection and freezing steps.
(DOC)

Table S4 **Cryopreservation process for multiple ejectors (4 ejectors).**
(DOC)

Table S5 **Cryopreservation process for multiple ejectors (25 ejectors).**
(DOC)

Table S6 Nonparametric Mann-Whitney U test results (p-values) of pairwise comparisons for ejection at two different distances (60 and 90 mm) and gas flow rates (3.2 and 4.8 l/min) for Cripps method(*) and Harboe method(**). Freezing was not affected from the ejection conditions as per nonparametric Kruskal-Wallis one way analysis of variance, therefore pairwise comparisons were not performed.
(DOC)

Table S7 **Appendix for symbols.**
(DOCX)
