# Supplementary figures and images for: Blood Banking in Living Droplets
Source: PLoS One. 2011 Mar 11;6(3):e17530. doi: 10.1371/journal.pone.0017530 (PMC3055869; doi:10.1371/journal.pone.0017530)

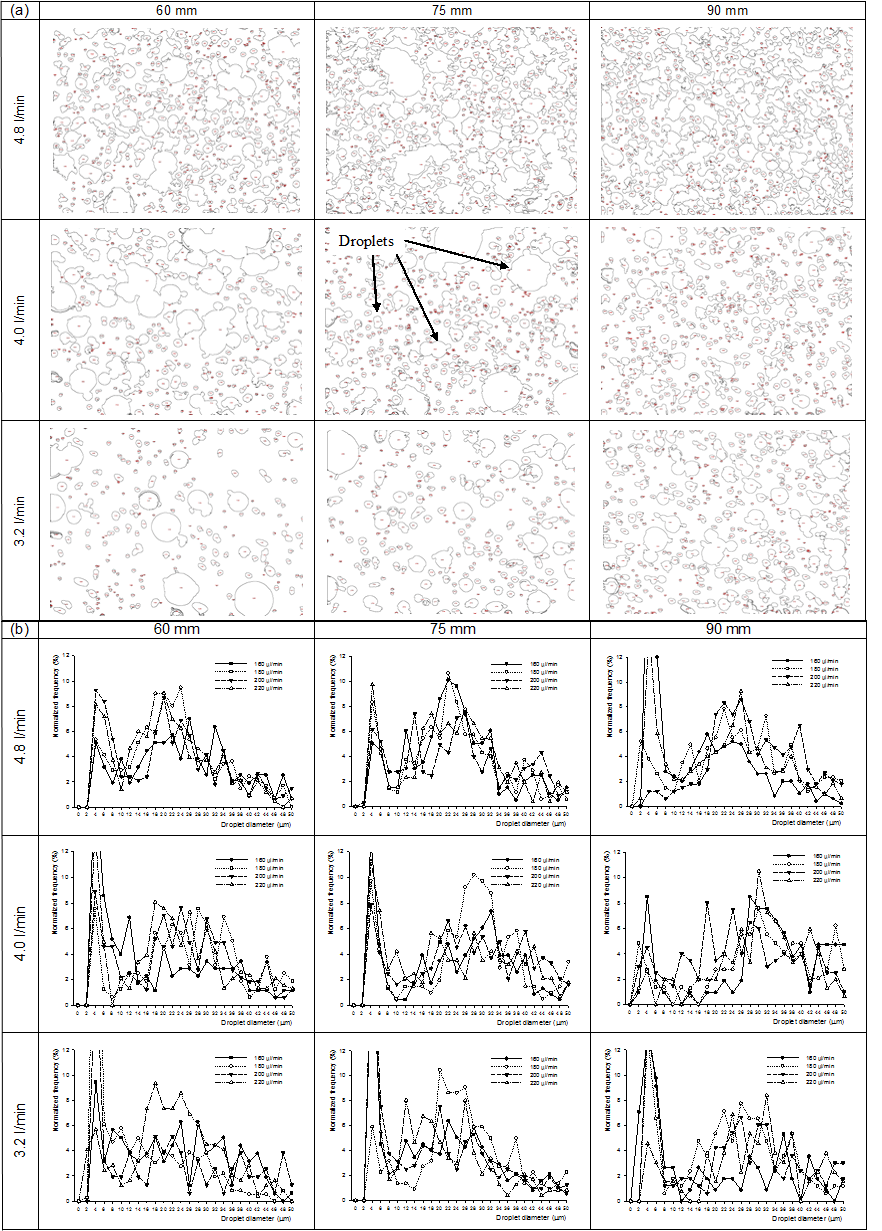

Supplement: Figure S2 — Droplet size measurements. (a) Images of ejected droplets on collection film. Outline tracking, measuring size, and counting of droplets using Image J software. Droplets were collected on paper with three different distances and sheath flow rates at a fixed blood flow rate, 200 µl/min. Droplet diameters were calculated using the area of each closed outline. (b) Droplet size distribution is shown as a function of droplet collecting distance, gas flow rate, and blood flow rate. (TIF) [file pone.0017530.s002.tif]
