# Supplementary material for: Blood Banking in Living Droplets
Source: PLoS One. 2011 Mar 11;6(3):e17530. doi: 10.1371/journal.pone.0017530 (PMC3055869; doi:10.1371/journal.pone.0017530)
Supplement: Table S1 — Composition of the cryoprotective solutions used (g/40 ml). (DOC) [file pone.0017530.s004.doc]

| CPA | Glycerol (ml) | Sorbitol (g) | NaCl (g) | Sterile DI Water (ml) |
| --- | --- | --- | --- | --- |
| 1.0 M | 2.9 | 1.16 | 0.25 | 37.1 |
| 2.0 M | 5.8 | 1.16 | 0.25 | 34.2 |
| 2.5 M | 7.3 | 1.16 | 0.25 | 32.7 |
| 4.0 M | 11.7 | 1.16 | 0.25 | 28.3 |
