# Supplementary material for: Blood Banking in Living Droplets
Source: PLoS One. 2011 Mar 11;6(3):e17530. doi: 10.1371/journal.pone.0017530 (PMC3055869; doi:10.1371/journal.pone.0017530)
Supplement: Table S2 — Spectrometer absorbance values for the two controls and actual sample from each step in the cryopreservation process from experimental conditions of 75 mm droplet collecting distance and 4.0 l/min of sheath gas flow rate). (DOC) [file pone.0017530.s005.doc]

| Absorbance | | Processes | | | | | Total % Hemolysis |
| --- | --- | --- | --- | --- | --- | --- | --- |
|  |  | CPA loading | | Cryopreservation | | |  |
|  |  | CPA_1_ | CPA_2_ | Ejection | Collection Film | Freezing |  |
| λ_416mm_ | ABS_0_ | 0.008 ± 0.001 | 0.051 ± 0.047 | 0.027 ± 0.021 | 0.700 ± 0.043 | 0.407 ± 0.045 |  |
|  | ABS_100_ | 3.849 ± 0.158 | 4.050 ± 0.187 | 1.833 ± 0.013 | 1.798 ± 0.061 | 1.124 ± 0.081 |  |
|  | ABS | 0.051 ± 0.047 | 0.122 ± 0.029 | 0.229 ± 0.020 | 0.631 ± 0.052 | 0.462 ± 0.053 |  |
| λ_545mm_ | ABS_0_ | -0.006 ± 0.003 | 0.006 ± 0.014 | -0.007 ± 0.017 | 0.098 ± 0.023 | 0.046 ± 0.012 |  |
|  | ABS_100_ | 0.447 ± 0.026 | 0.477 ± 0.019 | 0.205 ± 0.002 | 0.193 ± 0.007 | 0.119 ± 0.009 |  |
|  | ABS | 0.006 ± 0.014 | 0.037 ± 0.019 | 0.000 ± 0.011 | 0.079 ± 0.013 | 0.051 ± 0.013 |  |
| λ_576mm_ | ABS_0_ | -0.006 ± 0.002 | 0.006 ± 0.014 | -0.006 ± 0.016 | 0.103 ± 0.023 | 0.049 ± 0.012 |  |
|  | ABS_100_ | 0.486 ± 0.028 | 0.524 ± 0.021 | 0.223 ± 0.002 | 0.214 ± 0.008 | 0.132 ± 0.010 |  |
|  | ABS | 0.006 ± 0.014 | 0.037 ± 0.019 | 0.004 ± 0.011 | 0.084 ± 0.012 | 0.055 ± 0.012 |  |
| Cripps | ABS_0_ | 0.001 ± 0.000 | 0.004 ± 0.002 | 0.003 ± 0.001 | 0.047 ± 0.003 | 0.026 ± 0.003 |  |
|  | ABS_100_ | 0.291 ± 0.018 | 0.320 ± 0.015 | 0.134 ± 0.002 | 0.135 ± 0.005 | 0.085 ± 0.006 |  |
|  | ABS | 0.004 ± 0.002 | 0.007 ± 0.001 | 0.019 ± 0.001 | 0.041 ± 0.002 | 0.032 ± 0.002 |  |
| Harboe | ABS_0_ | 0.014 ± 0.003 | 0.042 ± 0.029 | 0.035 ± 0.008 | 0.552 ± 0.018 | 0.332 ± 0.031 |  |
|  | ABS_100_ | 3.167 ± 0.122 | 3.311 ± 0.159 | 1.517 ± 0.011 | 1.491 ± 0.050 | 0.931 ± 0.067 |  |
|  | ABS | 0.042 ± 0.029 | 0.079 ± 0.015 | 0.214 ± 0.005 | 0.532 ± 0.052 | 0.381 ± 0.038 |  |
| % Hemolysis | λ_416mm_ | 29.23 ± 0.91% | 1.79 ± 0.80% | 11.18 ± 0.39% | -6.29 ± 6.10% | 7.58 ± 2.51% | 43.49 ± 10.71% |
|  | λ_545mm_ | 2.65 ± 3.05% | 6.57 ± 4.37% | 3.61 ± 2.37% | -19.93 ± 23.36% | 5.94 ± 16.56% | -1.16 ± 52.71% |
|  | λ_576mm_ | 2.44 ± 2.80% | 5.92 ± 4.06% | 4.22 ± 2.06% | -17.07 ± 22.01% | 7.20 ± 12.65% | 2.71 ± 43.58% |
|  | Cripps | 0.92 ± 0.49% | 0.95 ± 0.11% | 11.83 ± 0.82% | -6.82 ± 4.68% | 8.88 ± 1.52% | 15.76 ± 7.61% |
|  | Harboe | 0.90 ± 0.88% | 1.13 ± 0.42% | 12.10 ± 0.46% | -2.09 ± 6.90% | 8.31 ± 1.13% | 20.35 ± 9.80% |
