# Supplementary material for: Blood Banking in Living Droplets
Source: PLoS One. 2011 Mar 11;6(3):e17530. doi: 10.1371/journal.pone.0017530 (PMC3055869; doi:10.1371/journal.pone.0017530)
Supplement: Table S3 — Percent hemolysis values of ejection, collection film, and freezing for five different experimental conditions are given. Total hemolysis is the sum of hemolysis due to the ejection and freezing steps. (DOC) [file pone.0017530.s006.doc]

| Distance (mm) | 60 | | | |  | 75 | | | |  | 90 | | | |  |
| --- | --- | --- | --- | --- | --- | --- | --- | --- | --- | --- | --- | --- | --- | --- | --- |
| Flow rate (l/min) | Methods | Ejection | Collection Film | Freezing | Total | Methods | Ejection | Collection Film | Freezing | Total | Methods | Ejection | Collection Film | Freezing | Total |
| 3.2 | λ_1, 416 nm_ | 7.92% | -7.01% | 8.27% |  |  |  |  |  |  | λ_1, 416 nm_ | 9.43% | -9.82% | 5.85% |  |
|  | λ_2, 545 nm_ | 9.26% | -5.91% | 48.31% |  |  |  |  |  |  | λ_2, 545 nm_ | 12.09% | -12.71% | 15.06% |  |
|  | λ_3, 576 nm_ | 8.88% | -5.77% | 44.74% |  |  |  |  |  |  | λ_3, 576 nm_ | 11.94% | -12.39% | 13.96% |  |
|  | Cripps | 6.87% | -6.38% | 5.20% | 14.07% |  |  |  |  |  | Cripps | 8.78% | -9.28% | 5.47% | 14.25% |
|  | Harboe | 7.46% | -1.63% | 3.85% | 11.31% |  |  |  |  |  | Harboe | 9.00% | -4.16% | 4.14% | 13.14% |
| 4.0 |  |  |  |  |  | λ_1, 416 nm_ | 11.18% | -6.29% | 7.58% |  |  |  |  |  |  |
|  |  |  |  |  |  | λ_2, 545 nm_ | 3.61% | -19.93% | 5.94% |  |  |  |  |  |  |
|  |  |  |  |  |  | λ_3, 576 nm_ | 4.22% | -17.07% | 7.20% |  |  |  |  |  |  |
|  |  |  |  |  |  | Cripps | 11.83% | -6.82% | 8.88% | 20.71% |  |  |  |  |  |
|  |  |  |  |  |  | Harboe | 12.10% | -2.09% | 8.31% | 20.51% |  |  |  |  |  |
| 4.8 | λ_1, 416 nm_ | 17.48% | -2.02% | 1.76% |  |  |  |  |  |  | λ_1, 416 nm_ | 15.97% | -5.36% | 6.05% |  |
|  | λ_2, 545 nm_ | 19.15% | -3.75% | 5.13% |  |  |  |  |  |  | λ_2, 545 nm_ | 13.81% | -4.83% | 13.60% |  |
|  | λ_3, 576 nm_ | 18.92% | -4.09% | 4.55% |  |  |  |  |  |  | λ_3, 576 nm_ | 13.72% | -4.96% | 11.98% |  |
|  | Cripps | 16.16% | -4.07% | 1.60% | 17.76% |  |  |  |  |  | Cripps | 14.74% | -6.74% | 3.02% | 17.76% |
|  | Harboe | 16.97% | -2.02% | 1.22% | 18.19% |  |  |  |  |  | Harboe | 16.36% | -2.31% | 4.02% | 20.38% |
