# Supplementary material for: Blood Banking in Living Droplets
Source: PLoS One. 2011 Mar 11;6(3):e17530. doi: 10.1371/journal.pone.0017530 (PMC3055869; doi:10.1371/journal.pone.0017530)
Supplement: Table S4 — Cryopreservation process for multiple ejectors (4 ejectors). (DOC) [file pone.0017530.s007.doc]

| Absorbance | | Cryopreservation | | | | | | Total % Hemolysis | | |
| --- | --- | --- | --- | --- | --- | --- | --- | --- | --- | --- |
|  |  | Ejection | | | Freezing | | |  |  |  |
| λ_416mm_ | ABS_0_ | 0.008 | ± | 0.001 | 0.373 | ± | 0.019 |  | | |
|  | ABS_100_ | 3.849 | ± | 0.158 | 1.861 | ± | 0.073 |  |  |  |
|  | ABS | 0.710 | ± | 0.051 | 0.416 | ± | 0.021 |  |  |  |
| λ_545mm_ | ABS_0_ | -0.006 | ± | 0.003 | 0.035 | ± | 0.007 |  |  |  |
|  | ABS_100_ | 0.447 | ± | 0.026 | 0.196 | ± | 0.011 |  |  |  |
|  | ABS | 0.105 | ± | 0.035 | 0.041 | ± | 0.011 |  |  |  |
| λ_576mm_ | ABS_0_ | -0.006 | ± | 0.002 | 0.038 | ± | 0.008 |  |  |  |
|  | ABS_100_ | 0.486 | ± | 0.028 | 0.217 | ± | 0.011 |  |  |  |
|  | ABS | 0.111 | ± | 0.034 | 0.043 | ± | 0.011 |  |  |  |
| Cripps | ABS_0_ | 0.001 | ± | 0.000 | 0.023 | ± | 0.002 |  |  |  |
|  | ABS_100_ | 0.291 | ± | 0.018 | 0.138 | ± | 0.005 |  |  |  |
|  | ABS | 0.048 | ± | 0.002 | 0.025 | ± | 0.002 |  |  |  |
| Harboe | ABS_0_ | 0.014 | ± | 0.003 | 0.315 | ± | 0.016 |  |  |  |
|  | ABS_100_ | 3.167 | ± | 0.122 | 1.542 | ± | 0.056 |  |  |  |
|  | ABS | 0.561 | ± | 0.026 | 0.344 | ± | 0.009 |  |  |  |
| % Hemolysis | λ_416mm_ | 18.28% | ± | 2.03% | 2.85% | ± | 2.63% | 21.13% | ± | 4.66% |
|  | λ_545mm_ | 24.54% | ± | 8.64% | 3.73% | ± | 8.83% | 28.28% | ± | 17.47% |
|  | λ_576mm_ | 23.70% | ± | 7.68% | 2.79% | ± | 8.62% | 26.49% | ± | 16.30% |
|  | Cripps | 16.12% | ± | 1.66% | 1.45% | ± | 1.90% | 17.58% | ± | 3.56% |
|  | Harboe | 17.35% | ± | 1.47% | 2.40% | ± | 1.82% | 19.76% | ± | 3.29% |
