# Supplementary material for: Blood Banking in Living Droplets
Source: PLoS One. 2011 Mar 11;6(3):e17530. doi: 10.1371/journal.pone.0017530 (PMC3055869; doi:10.1371/journal.pone.0017530)
Supplement: Table S5 — Cryopreservation process for multiple ejectors (25 ejectors). (DOC) [file pone.0017530.s008.doc]

| Absorbance | | Cryopreservation | | | | | | Total % Hemolysis | | |
| --- | --- | --- | --- | --- | --- | --- | --- | --- | --- | --- |
|  |  | Ejection | | | Freezing | | |  |  |  |
| λ_416mm_ | ABS_0_ | 0.066 | ± | 0.005 | 0.534 | ± | 0.043 |  | | |
|  | ABS_100_ | 1.562 | ± | 0.027 | 4.050 | ± | 0.187 |  |  |  |
|  | ABS | 0.334 | ± | 0.030 | 0.582 | ± | 0.016 |  |  |  |
| λ_545mm_ | ABS_0_ | 0.014 | ± | 0.002 | 0.057 | ± | 0.004 |  |  |  |
|  | ABS_100_ | 0.164 | ± | 0.003 | 0.477 | ± | 0.019 |  |  |  |
|  | ABS | 0.051 | ± | 0.038 | 0.068 | ± | 0.011 |  |  |  |
| λ_576mm_ | ABS_0_ | 0.014 | ± | 0.002 | 0.063 | ± | 0.005 |  |  |  |
|  | ABS_100_ | 0.181 | ± | 0.003 | 0.524 | ± | 0.021 |  |  |  |
|  | ABS | 0.053 | ± | 0.036 | 0.075 | ± | 0.010 |  |  |  |
| Cripps | ABS_0_ | 0.004 | ± | 0.001 | 0.038 | ± | 0.004 |  |  |  |
|  | ABS_100_ | 0.117 | ± | 0.001 | 0.320 | ± | 0.015 |  |  |  |
|  | ABS | 0.022 | ± | 0.001 | 0.043 | ± | 0.003 |  |  |  |
| Harboe | ABS_0_ | 0.048 | ± | 0.005 | 0.446 | ± | 0.036 |  |  |  |
|  | ABS_100_ | 1.305 | ± | 0.024 | 3.311 | ± | 0.159 |  |  |  |
|  | ABS | 0.259 | ± | 0.014 | 0.474 | ± | 0.024 |  |  |  |
| % Hemolysis | λ_416mm_ | 17.91% | ± | 1.99% | 1.36% | ± | 1.27% | 19.27% | ± | 3.26% |
|  | λ_545mm_ | 24.67% | ± | 24.43% | 2.70% | ± | 3.62% | 27.36% | ± | 28.05% |
|  | λ_576mm_ | 23.51% | ± | 20.91% | 2.67% | ± | 3.12% | 26.18% | ± | 24.04% |
|  | Cripps | 16.25% | ± | 1.39% | 1.83% | ± | 1.20% | 18.08% | ± | 2.59% |
|  | Harboe | 16.80% | ± | 1.15% | 0.97% | ± | 1.18% | 17.77% | ± | 2.33% |
