# Supplementary material for: Blood Banking in Living Droplets
Source: PLoS One. 2011 Mar 11;6(3):e17530. doi: 10.1371/journal.pone.0017530 (PMC3055869; doi:10.1371/journal.pone.0017530)
Supplement: Table S6 — Nonparametric Mann-Whitney U test results (p-values) of pairwise comparisons for ejection at two different distances (60 and 90 mm) and gas flow rates (3.2 and 4.8 l/min) for Cripps method(*) and Harboe method(**). Freezing was not affected from the ejection conditions as per nonparametric Kruskal-Wallis one way analysis of variance, therefore pairwise comparisons were not performed. (DOC) [file pone.0017530.s009.doc]

| Mann-Whitney U test | 60/3.2 - 60/4.8 | 90/3.2 - 90/4.8 | 60/3.2 - 90/3.2 | 60/4.8 - 90/4.8 |
| --- | --- | --- | --- | --- |
| Cripps method(*) | P_1_ | P_2_ | P_3_ | P_4_ |
| Ejection | 0.04 | 0.04 | 0.26 | 0.33 |
| Freezing | - | - | - | - |
| Harboe method(**) | P_1_ | P_2_ | P_3_ | P_4_ |
| Ejection | 0.04 | 0.04 | 0.26 | 0.33 |
| Freezing | - | - | - | - |
