# Supplementary material for: Blood Banking in Living Droplets
Source: PLoS One. 2011 Mar 11;6(3):e17530. doi: 10.1371/journal.pone.0017530 (PMC3055869; doi:10.1371/journal.pone.0017530)
Supplement: Table S7 — Appendix for symbols. (DOCX) [file pone.0017530.s010.docx]

| WB | Whole blood |
| --- | --- |
| RBC | Red blood cell |
| CPA | Cryoprotective agent |
| CPDA-1 | Citrate phosphate dextrose adenine |
| DPBS | Dulbecco’s Phosphate Buffered Saline |
| CPA_1_ | Mixture of RBCs and final 1M glycerol concentration after first CPA loading step |
| CPA_2_ | Mixture of RBCs and final 2.5M glycerol concentration after second CPA loading step |
| $\mathrm{ABS}_{0}$ | Absorbance of free hemoglobin before each process |
| $\mathrm{ABS}_{100}$ | Absorbance of free hemoglobin with 100% hemolysis |
| $\mathrm{ABS}_{\mathrm{process}}$ | Absorbance of free hemoglobin after each process |
| $\mathrm{ABS}_{0\_\mathrm{CPA}_{1}}$ | Absorbance of free hemoglobin before loading CPA1 |
| $\mathrm{ABS}_{100\_\mathrm{CPA}_{1}}$ | Absorbance of free hemoglobin before loading CPA1 with 100% hemolysis |
| $\mathrm{ABS}_{\mathrm{CPA}_{1}}$ | Absorbance of free hemoglobin after loading CPA1 |
| $\mathrm{ABS}_{0\_\mathrm{CPA}_{2}}$ | Absorbance of free hemoglobin before loading CPA2 |
| $\mathrm{ABS}_{100\_\mathrm{CPA}_{2}}$ | Absorbance of free hemoglobin before loading CPA2 with 100% hemolysis |
| $\mathrm{ABS}_{\mathrm{CPA}_{2}}$ | Absorbance of free hemoglobin after loading CPA2 |
| $\mathrm{ABS}_{0\_ejection}$ | Absorbance of free hemoglobin before ejection |
| $\mathrm{ABS}_{100\_ejection}$ | Absorbance of free hemoglobin before ejection with 100% hemolysis |
| $\mathrm{ABS}_{\mathrm{ejection}}$ | Absorbance of free hemoglobin after ejection |
| $\mathrm{ABS}_{0\_film}$ | Absorbance of free hemoglobin ejected into 2.5 M glycerol solution |
| $\mathrm{ABS}_{100\_film}$ | Absorbance of free hemoglobin ejected into DI water |
| $\mathrm{ABS}_{\mathrm{film}}$ | Absorbance of free hemoglobin ejected onto collection film |
| $\mathrm{ABS}_{0\_fs}$ | Absorbance of free hemoglobin ejected onto collection film water and then dipping into 2.5 M glycerol |
| $\mathrm{ABS}_{100\_fs}$ | Absorbance of free hemoglobin ejected onto collection film and then dipping into DI |
| $\mathrm{ABS}_{\mathrm{fs}}$ | Absorbance of free hemoglobin ejected onto PE film and then freeze and thaw into 2.5 M glycerol |
| $\mathrm{ABS}_{0\_ejector\_x25}$ | Absorbance of free hemoglobin before ejection using 25 ejector system |
| $\mathrm{ABS}_{100\_ejector\_x25}$ | Absorbance of free hemoglobin before ejection with 100% hemolysis using 25 ejector system |
| $\mathrm{ABS}_{ejector\_x25}$ | Absorbance of free hemoglobin after ejection using 25 ejector system |
| $\mathrm{ABS}_{0\_film\_x25}$ | Absorbance of free hemoglobin ejected into 2.5 M glycerol solution using 25 ejector system |
| $\mathrm{ABS}_{100\_film\_x25}$ | Absorbance of free hemoglobin ejected into DI water using 25 ejector system |
| $\mathrm{ABS}_{film\_x25}$ | Absorbance of free hemoglobin ejected onto collection film using 25 ejector system |
| $\mathrm{ABS}_{0\_fs\_x25}$ | Absorbance of free hemoglobin ejected onto collection film water and then immersing into 2.5 M glycerol using 25 ejector system |
| $\mathrm{ABS}_{100\_fs\_x25}$ | Absorbance of free hemoglobin ejected onto collection film and then immersing into DI using 25 ejector system |
