# Supplementary material for: Blood Banking in Living Droplets
Source: PLoS One. 2011 Mar 11;6(3):e17530. doi: 10.1371/journal.pone.0017530 (PMC3055869; doi:10.1371/journal.pone.0017530)
Supplement: Supplementary file 9 [file pone.0017530.s003.docx]

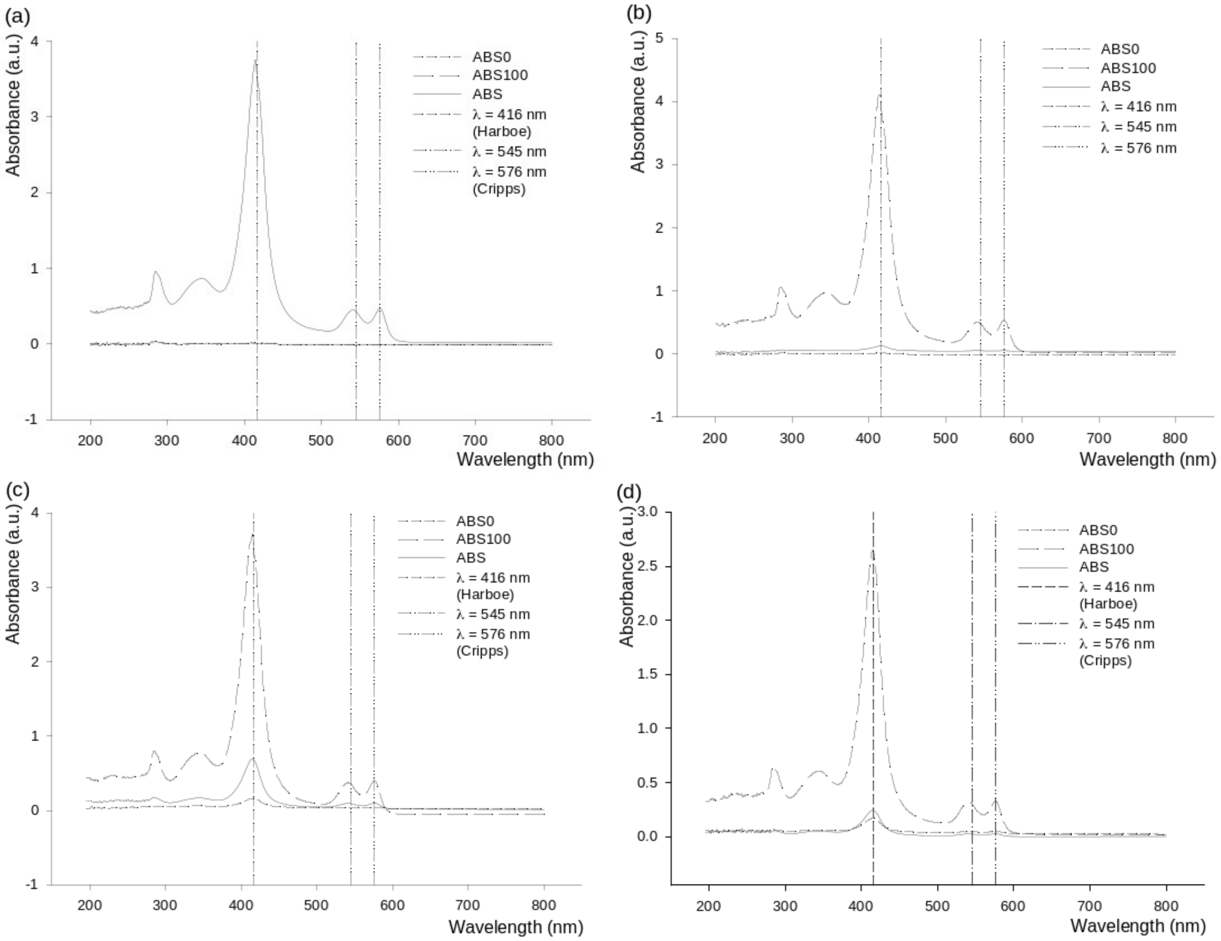
(a)
Absorbance (a.u.)
Wavelength (nm)
ABS0
ABS100
ABS
λ = 416 nm (Harboe)
λ = 545 nm
λ = 576 nm (Cripps)
(b)
Absorbance (a.u.)
Wavelength (nm)
ABS0
ABS100
ABS
λ = 416 nm
λ = 545 nm
λ = 576 nm
(c)
Absorbance (a.u.)
Wavelength (nm)
ABS0
ABS100
ABS
λ = 416 nm (Harboe)
λ = 545 nm
λ = 576 nm (Cripps)
(d)
Absorbance (a.u.)
Wavelength (nm)
ABS0
ABS100
ABS
λ = 416 nm (Harboe)
λ = 545 nm
λ = 576 nm (Cripps)
